# Impact of heart failure severity on the mortality benefit of mitral transcatheter edge-to-edge valve repair

**DOI:** 10.1007/s00392-024-02490-7

**Published:** 2024-07-24

**Authors:** Valeria Magni, Marianna Adamo, Elisa Pezzola, Antonio Popolo Rubbio, Cristina Giannini, Giulia Masiero, Carmelo Grasso, Paolo Denti, Arturo Giordano, Federico De Marco, Antonio L. Bartorelli, Matteo Montorfano, Cosmo Godino, Cesare Baldi, Francesco De Felice, Annalisa Mongiardo, Ida Monteforte, Emmanuel Villa, Gabriele Crimi, Maurizio Tusa, Luca Testa, Lisa Serafini, Dario Cani, Giacinta Guarini, Alda Huqi, Marco Sesana, Marco De Carlo, Francesco Maisano, Giuseppe Tarantini, Corrado Tamburino, Francesco Bedogni, Marco Metra

**Affiliations:** 1 Division of Cardiology and Cardiac Catheterization Laboratory, ASST Spedali Civili di Brescia and Department of Medical and Surgical Specialties, Radiological Sciences, and Public Health, University of Brescia, Brescia, Italy; 2 Division of Cardiology, Ospedale di Desenzano, Desenzano, Italy; 3 Department of Cardiology, IRCCS Policlinico San Donato, San Donato Milanese, Milan, Italy; 4 Cardiac Catheterization Laboratory, Cardiothoracic and Vascular Department, Azienda Ospedaliero Universitaria Pisana, Pisa, Italy; 5 Interventional Cardiology Unit, Department of Cardiac, Thoracic and Vascular Science, University of Padua, Padua, Italy; 6 Division of Cardiology, Centro Alte Specialità e Trapianti (CAST), Azienda Ospedaliero-Universitaria Policlinico-Vittorio Emanuele, University of Catania, Catania, Italy; 7 Cardiac Surgery Department, San Raffaele University Hospital, Milan, Italy; 8 Invasive Cardiology Unit, Pineta Grande Hospital, Castel Volturno, Caserta, Italy; 9 Centro Cardiologico Monzino, IRCCS, Milan, Italy; 10 Interventional Cardiology Unit IRCCS San Raffaele Scientific Institute, Milan, Italy; 11 Hospital San Giovanni di Dio e Ruggi d’Aragona, Salerno, Italy; 12 Division of Interventional Cardiology, Azienda Ospedaliera S. Camillo Forlanini, Rome, Italy; 13 Division of Cardiology, University Magna Graecia, Catanzaro, Italy; 14 AORN Ospedali dei Colli, Monaldi Hospital, Naples, Italy; 15 Cardiac Surgery Unit Poliambulanza Hospital, Fondazione Poliambulanza, Brescia, Italy; 16 Cardiac Catheterization Laboratory, Policlinico San Martino, Genoa, Italy; 17 Cardiac Catheterization Laboratory, Policlinico San Matteo, Pavia, Italy; 18 Division of Cardiology, Cardiothoracic and Vascular Department, Azienda Ospedaliero Universitaria Pisana, Pisa, Italy

**Keywords:** Secondary mitral regurgitation, Mitral transcatheter edge-to-edge repair, Advanced heart failure

## Abstract

**Background:**

To assess the interaction between heart failure (HF) severity and optimal reduction of secondary mitral regurgitation (SMR) on mortality in patients undergoing transcatheter edge-to-edge repair (M-TEER).

**Methods and results:**

Among 1656 patients included in the Italian Society of Interventional Cardiology (GIse) registry Of Transcatheter treatment of mitral valve regurgitaTiOn (GIOTTO) 984 had SMR and complete data on advanced HF. Advanced HF was defined as NYHA class III or IV, left ventricular ejection fraction ≤ 30%, and > 1 HF hospitalization during the last 12 months. Optimal M-TEER was defined as residual SMR ≤ 1 + at discharge. One hundred sixteen patients (11.8%) had advanced HF. Achievement of an optimal SMR reduction was similar in patients with and without advanced HF (65% and 60% respectively). Advanced HF was an independent predictor of 2-year all-cause death (adjusted HR 1.52, 95% CI 1.09–2.10). Optimal M-TEER, as compared to a no-optimal M-TEER, was associated with a reduced risk of death both in patients with advanced (HR 0.55, 95% CI 0.32–0.97; *p* = 0.039) and no-advanced HF (HR 0.59, 95% CI 0.46–0.78; *p* < 0.001; *p* = 0.778 for interaction).

**Conclusions:**

Advanced HF is associated with poor outcome in patients undergoing M-TEER. However, an optimal SMR reduction reduces the risk of 2-year mortality regardless of HF severity.

**Graphical Abstract:**

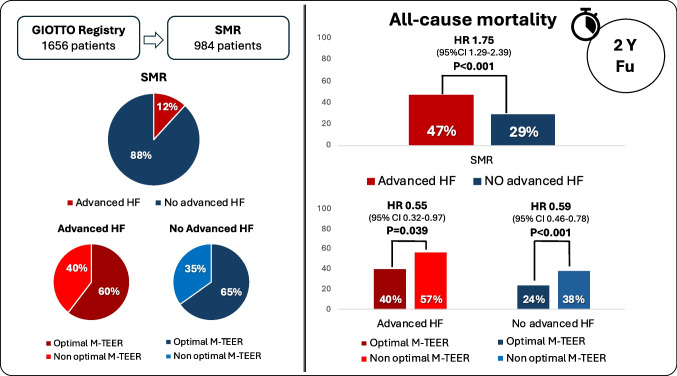

**Supplementary Information:**

The online version contains supplementary material available at 10.1007/s00392-024-02490-7.

## Introduction

Secondary mitral regurgitation (SMR) is common in patients with chronic heart failure (HF) and it is known to be associated with poor outcome [1, 2]. Current guidelines recommend mitral transcatheter edge-to-edge repair (M-TEER), with a class IIa recommendation, for HF patients with significant SMR and the specific clinical and echocardiographic characteristic [3, 4] of the patients enrolled in the Cardiovascular Outcomes Assessment of the MitraClip Percutaneous Therapy for Heart Failure Patients With Functional Mitral Regurgitation (COAPT) trial [5, 6]. Conversely, the indication to M-TEER is currently IIb, as an alternative to palliative care or as a bridge to other therapies, for the patients with HF who do not fulfill the COAPT criteria [3]. Notably, advanced HF was a major exclusion criterion in COAPT.

Nevertheless, some registries reported a possible beneficial effect of M-TEER in the setting of advanced HF showing both quality of life and symptoms improvement after the procedure [7–14].

Furthermore, M-TEER has been reported as a possible bridge strategy to left ventricular assist device or heart transplantation with a relevant proportion of patients improving their hemodynamic conditions after SMR reduction [15, 16]. However, it is currently unknown whether M-TEER may have an impact on mortality in patients with advanced HF.

The aim of this analysis is to explore the role of advanced HF in patients undergoing M-TEER as well as the association between an optimal reduction of SMR by means of M-TEER and mortality in patients with and without advanced HF.

## Methods

### Population and definitions

The multicenter Italian Society of Interventional Cardiology (GIse) registry Of Transcatheter treatment of mitral valve regurgitaTiOn (GIOTTO) is a single-arm, multicenter, prospective registry conceived to collect data regarding patients with symptomatic mitral regurgitation (MR) who underwent MitraClip between 2016 and 2020, reflecting standard clinical practice in Italian hospitals. Qualifying inclusion and exclusion criteria, echocardiographic selection and protocols employed, and details of the MitraClip procedure have been reported previously [17].

For the purpose of the present analysis, we only included patients with SMR and available data on advanced HF. The population of interest was stratified according to the presence of advanced HF.

Based on recent recommendations, advanced HF was defined according to the following criteria (all must be fulfilled): NYHA functional class III or IV, LVEF ≤ 30%, and > 1 hospitalization for HF during the last 12 months [3, 18]. Of note, information regarding baseline functional status (i.e., 6 min walking test and pulmonary exercise test) was not available in the GIOTTO registry.

Optimal M-TEER was defined as mild or less residual SMR (MR ≤ 1 +) assessed at discharge.

Outcome of interest was all-cause mortality assessed at 2-year follow-up. Additional outcomes were HF hospitalization and the composite of all-cause mortality and HF hospitalization.

### Statistical analysis

Categorical and dichotomous variables were expressed as absolute numbers and percentages and were compared by the chi-square test. Continuous variables were expressed as mean ± standard deviation, or median and interquartile range (25th–75th IQR), as appropriate. Unpaired Student’s *t*-test was used to compare continuous parameters following a normal distribution, while the Mann–Whitney *U* test was used to compare continuous variables with skewed distribution.

Two-year follow-up Kaplan–Meier curves for all-cause mortality, HF hospitalization, and the composite of all-cause mortality and HF hospitalization were computed after stratification by the presence of advanced HF. Comparisons were made with the log-rank test.

Univariate and multivariate Cox proportional hazards regression was used to assess the association between advanced HF and outcomes and to estimate corresponding hazard ratios (HR) and 95% confidence intervals (CI).

Variables included in the multivariable model were EuroSCORE II, chronic obstructive pulmonary disease (COPD), coronary artery disease (CAD), glomerular filtration rate (GFR), left ventricle end-systolic volume (LVESV), tricuspid annular plane systolic excursion (TAPSE), and optimal M-TEER.

Interaction between optimal M-TEER and advanced HF on outcomes was tested with a Cox regression analysis and reported as *p* for interaction.

For all analyses, the SPSS statistics software (version 25, IBM Corp., Armonk, NY, USA) was used and a *p*-value of ≤ 0.05 was set for significance.

## Results

### Baseline and procedural data

**Fig. 1:**
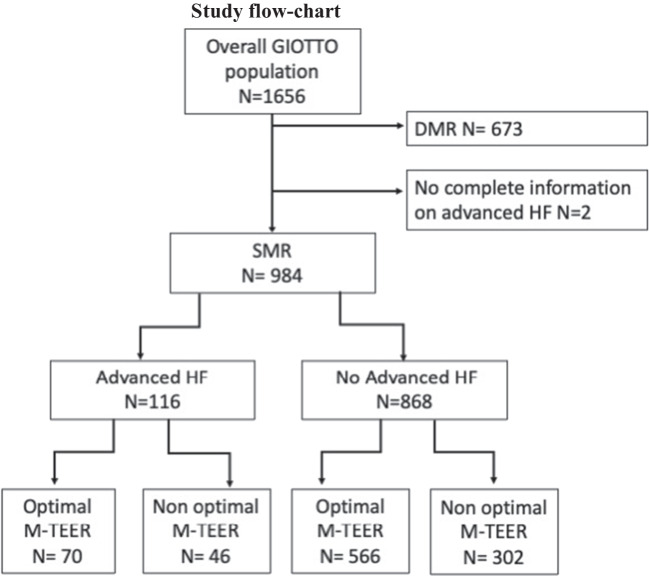
Number of patients included in the analysis and number of patients per subgroup are reported

**Fig. 2:**
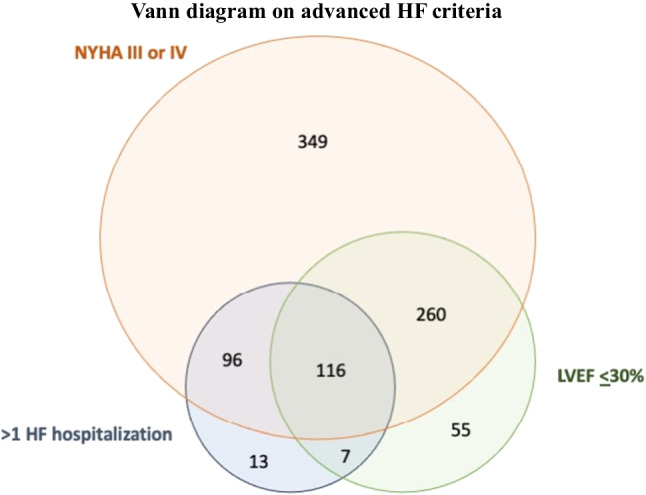
Number of patients fulfilling the different criteria is reported. Notably, 116 patients fulfilled all criteria and were considered having advanced HF

Among 1656 patients included in the GIOTTO registry, 984 had SMR and complete information on advanced HF Among them, 116 (11.8%) were classified as having advanced HF and 868 (88.2%) as having no-advanced HF (Fig. 1). Overall, 896 (91%) of patients had at least 1 criterion included in the definition of advanced HF. Distribution of advanced HF criteria is reported in Fig. 2. Baseline characteristics of the overall population, and stratified by the presence of advanced HF, are reported in Table 1.

**Table 1:** Baseline characteristics

	Overall (*N* = 984)	Advanced HF (*N* = 116)	No-advanced HF (*N* = 868)	*p-*value
Age [yrs], mean (± SD)	73.1 (± 8.6)	72.0 (± 7.9)	73.3 (± 8.7)	0.05
Sex [male], *N* (%)	622 (63.2%)	73 (62.9%)	549 (63.2%)	0.95
BMI, mean (± SD)	25.4 (± 4.0)	24.6 (± 3.9)	25.5 (± 4.0)	0.026
Diabete mellitus, *N* (%)	331 (33.6%)	42 (36.2%)	298 (33.3%)	0.53
eGFR [mL/m^2^], mean (± SD)	48.9 (± 24.5)	45.2 (± 23.8)	49.5 (± 24.5)	0.04
COPD, *N* (%)	142 (14.4%)	26 (22.4%)	116 (13.4%)	0.009
AF, *N* (%)	491 (49.9%)	66 (56.9%)	425 (49.0%)	0.11
CAD, *N* (%)	542 (55.1%)	69 (59.5%)	473 (54.5%)	0.31
Previous stroke, *N* (%)	81 (8.2%)	14 (12.1%)	67 (7.7%)	0.11
EuroSCORE II, mean (± SD)	7.7 (± 6.7)	9.6 (± 7.8)	7.4 (± 6.4)	< 0.001
Echocardiographic findings				
LVEDV [mL], mean (± SD)	176.6 (± 66.3)	207.4 (± 68.2)	172.3 (± 64.9)	< 0.001
LVEF [%], mean (± SD)	33.8 (± 10.0)	25.0 (± 4.3)	35.0 (± 10.0)	< 0.001
sPAP [mmHg], mean (± SD)	47.4 (± 13.5)	49.2 (± 15.0)	47.2 (± 13.3)	0.196
TAPSE [mm], mean (± SD)	17.7 (± 4.3)	16.1 (± 3.8)	17.9 (± 4.4)	0.001
MR 4 + , *N* (%)	742 (75.4%)	83 (71.6%)	659 (75.9%)	0.31
TR 4 + , *N* (%)	119 (12.1%)	15 (12.9%)	104 (12.0%)	0.77
Medical therapy				
Beta-blockers, *N* (%)	817 (83.0%)	100 (86.2%)	717 (82.6%)	0.33
ACE-I/ARB/ARNI, *N* (%)	325 (33.0%)	42 (36.2%)	283 (32.6%)	0.44
MRA, *N* (%)	542 (55.1%)	78 (67.2%)	464 (53.5%)	0.005
Loop diuretic, *N* (%)	918 (93.3%)	114 (98.3%)	804 (92.6%)	0.02

*ACE‐I*, angiotensin-converting enzyme inhibitor; *AF*, atrial fibrillation; *ARB*, angiotensin II receptor blocker; *ARNI*, angiotensin receptor‐neprilysin inhibitor; *BMI*, body mass index; *CAD*, coronary artery disease; *COPD*, chronic obstructive pulmonary disease; *eGFR*, estimated glomerular filtration rate; *HF*, heart failure; LVEDV, left ventricular end-diastolic volume; *LVEF*, left ventricular ejection fraction; *MR*, mitral regurgitation; *MRA*, mineralocorticoid receptor antagonist; *SD*, standard deviation; *sPAP*, systolic pulmonary artery pressure; *TAPSE*, tricuspid annular plane systolic excursion; *TR*, tricuspid regurgitation

Patients with advanced HF were younger and with a lower body mass index (BMI) as compared to those without advanced HF. In addition, they were more likely to have chronic obstructive pulmonary disease (COPD), higher EuroSCORE II, poorer kidney function, larger left ventricle, and worse left and right ventricular function, compared with the others. Regarding medical therapy, there were no differences in the proportion of patients receiving renin-angiotensin system antagonists or beta-blockers. However, compared with patients with no-advanced HF, those with advanced HF more frequently received loop diuretics and mineralocorticoid receptor antagonist (MRA).

Procedural and discharge data are shown in Table 2. An optimal SMR reduction, assessed at discharge, was achieved in 60% of patients with advanced HF and in 65% of patients with no-advanced HF (*p* = 0.304) (Table 2 and Fig. 1). Procedural time as well as device time was longer in patients with as compared to those without advanced HF. No differences were noted between the two groups in terms of number of clips deployed and mean pressure gradient at discharge.

**Table 2:** Procedural and discharge data

	Overall (*N* = 984)	Advanced HF (*N* = 116)	No-advanced HF (*N* = 868)	*p*-value
Number of clip (mean ± SD)	1.73 ± 0.7	1.84 ± 0.8	1.71 ± 0.6	0.053
Device time, min (mean ± SD)	65.4 ± 40.7	78.7 ± 55.1	63.5 ± 38.1	0.005
Procedural time, min (mean ± SD)	147.3 ± 69.7	169.6 ± 82.5	144.3 ± 67.2	0.002
Residual MR ≤ 1 + , *N* (%)	637 (64.6%)	70 (60%)	566 (65%)	0.304
Mean gradient, mmHg (mean ± SD)	3.5 ± 1.6	3.38 ± 1.46	3.47 ± 1.63	0.587

*HF*, heart failure; *MR*, mitral regurgitation; *SD*, standard deviation

### Outcomes

**Fig. 3:**
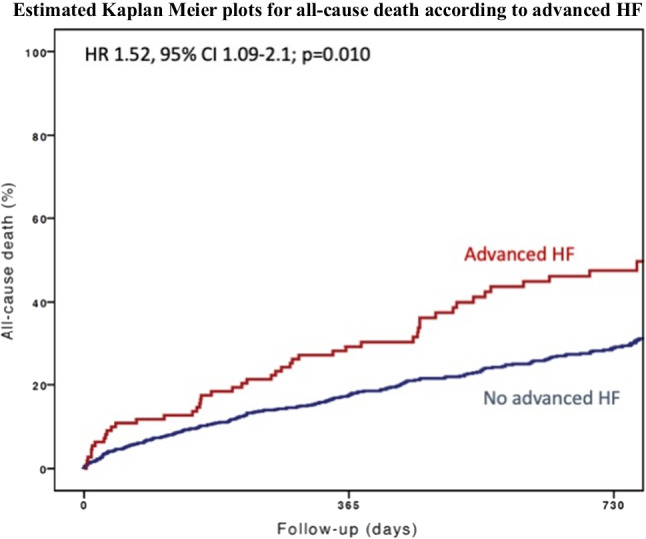
Cumulative incidence of 2-year all-cause death in patients with and without advanced HF

**Fig. 4:**
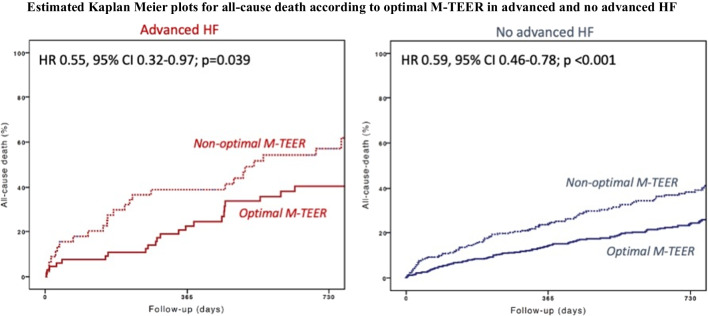
Cumulative incidence of 2-year all-cause death in patients who received or not an optimal M-TEER, in advanced HF (red plots) and no-advanced (blue plots)

Median follow-up was 614 days (IQ 317–763). At 2 years, 268 patients died. As expected, cumulative incidence of all-cause death was higher in patients with advanced HF versus those without advanced HF (47% vs 29%) (Fig. 3). Advanced HF was a predictor of death at both univariate (HR 1.75, 95% CI 1.29–2.39; *p* < 0.001) and multivariable (HR 1.52, 95% CI 1.09–2.1; *p* = 0.010) analyses. An optimal M-TEER was associated with a lower cumulative incidence of 2-year all-cause death, as compared to a no-optimal M-TEER, in both advanced (40% vs 57%; HR 0.55, 95% CI 0.32–0.97; *p* = 0.039) and non-advanced (24% vs 38%; HR 0.59, 95% CI 0.46–0.78; *p* < 0.001) HF patients (Fig. 4). Advanced HF did not affect the association between optimal M-TEER and all-cause death (*p* for interaction, 0.736).

Likewise, advanced HF was associated with higher incidence of HF hospitalization (HR 2.085, CI 1.393–3.120, *p* < 0.001) and of the composite outcome of all-cause mortality and HF hospitalization (HR 2.080, CI 1.529–2.829, *p* < 0.001) (Supplementary Figs. 1 and 2). An optimal M-TEER was associated with a lower incidence of HF hospitalization (HR 0.555, CI 0.392–0.784, *p* = 0.001) and of all-cause mortality and HF hospitalization (HR 0.603, CI 0.461–0.788, *p* < 0.001) in non-advanced HF (Supplementary Figs. 3 and 4), but not in advanced HF patients (HR 0.969, CI 0.457–2.054, *p* = 0.934 and HR 0.627, CI 0.379–1.040, *p* = 0.070 respectively) (Supplementary Figs. 5 and 6). However, advanced HF did not affect the association between optimal M-TEER and additional outcomes (*p* for interaction 0.171 for HF hospitalization, and 0.778 for all-cause mortality and HF hospitalization).

## Discussion

The main findings of the present study are the following: i) among patients with SMR undergoing M-TEER in the GIOTTO multicenter registry, those with advanced HF had a higher risk of mortality and, ii) an optimal M-TEER was associated with a lower risk of mortality, as compared to a no-optimal M-TEER, in both patients with and without advanced HF.

M-TEER is a well-established therapy for patients with significant isolated SMR and favorable clinical and echocardiographic characteristics, as in the COAPT Trial [6, 19, 20]. However, a high proportion of real-world patients has a non-COAPT profile [13, 21] and, despite the expanded use of M-TEER also in this setting, [22] evidence supporting this indication is limited. Specifically, some registries reported safety and feasibility of M-TEER in non-COAPT patients as well as in patients with advanced or severe HF with an improvement of hemodynamic parameters as well as symptoms, functional status, and quality of life after the procedure [7, 10, 16]. However, a comparison group (i.e., patients who did not receive the intervention) was missing in previous studies and data regarding the effective prognostic benefit of M-TEER in patients with advanced HF are lacking.

Also, data regarding the natural history and prognostic role of severe SMR in the setting of advanced HF are limited. A recent study showed that severe SMR was associated with an increased risk of cardiovascular mortality and recurrent HF hospitalization, but not all-cause mortality in patients with advanced HF [23]. Furthermore, in current guidelines, the hypothetical goal of the indication for M-TEER in non-COAPT patients is the improvement of HF symptoms alone.

### Prognostic role of advanced HF in M-TEER

Several registries underlined the negative prognostic role of variables related to advanced HF in patients undergoing M-TEER [7, 24–27]. Predictors of 1-year mortality in the German multicenter TRAnscatheter Mitral valve Interventions (TRAMI) registry were NYHA class IV (HR 1.62), anemia (HR 2.44), renal failure with serum creatinine ≥ 1.5 mg/dL (HR 1.77), LVEF < 30% (HR 1.59), and severe tricuspid regurgitation (HR 1.84) [28]. In this registry, among 777 patients undergoing MitraClip, 256 (33.0%) had a LVEF < 30% [11, 28]. Multivariable analysis revealed an increased risk of major adverse events (mainly driven by all-cause death) in patients with severely impaired LV function. Advanced NYHA class, especially NYHA IV, was extensively reported as associated with poorer outcome even after M-TEER [27–29]. Franzen et al. [7] showed that among patients with SMR and advanced HF, NYHA class, elevated NTpro-BNP levels, and LV dimensions were major predictors of adverse outcome [7]. Recurrent (> 1) HF hospitalizations before M-TEER have also been reported as associated with unfavorable outcome after the procedure [30].

We confirm all these previous findings showing that advanced HF, defined as NYHA III or IV, LVEF ≤ 30%, and > 1 HF hospitalization, was associated with an increased risk of 2-year all-cause mortality (regardless of possible confounders), HF hospitalization, and the composite of all-cause mortality and HF hospitalization. Notably, right ventricular (RV) function has a crucial prognostic role in HF patients with SMR undergoing M-TEER [12, 31–33]. In our analysis, according to recent recommendations, RV function was not included in the definition of advanced HF. However, as expected, RV dysfunction (i.e. a low TAPSE) was more frequent in patients with advanced versus no-advanced HF.

### Prognostic role of optimal M-TEER

Among patients with HF and severe SMR enrolled in the COAPT trial, a favorable outcome at 12-month follow-up was predicted only by lower serum creatinine, Kansas City Cardiomyopathy Questionnaire overall summary score (KCCQ-OS), and treatment assignment to M-TEER plus GDMT arm, corroborated by the 30-day change in MR, suggesting that the mechanism underlying clinical response to M-TEER is reduction of MR. In comparison with residual MR ≤ 1, MR 2 + demonstrated a trend towards mortality benefit at both unadjusted (HR, 1.34; 95% CI, 0.99 to 1.82; *p* = 0.056) and adjusted (HR, 1.35; 95% CI, 0.99 to 1.84; *p* = 0.057) analyses [34].

The association between an optimal MR reduction by M-TEER and improved survival in SMR patients has already been extensively reported [26, 35]. The Optimized Catheter Valvular Intervention (OCEAN-Mitral) registry, a prospective, multicenter registry to assess the safety and efficacy of TEER in significant MR, enrolled 2150 consecutive, symptomatic patients who underwent M-TEER in 21 Japanese institutions [36]. Among them, 1617 patients (75.2%) were deemed to have SMR. Looking at the single components that define advance HF, 63.2% of patients had a NYHA functional class III or IV, 71.7% HF experienced > 1 hospitalization within 1 year before TEER, but only 22.7% of them had an LVEF < 30%. Mortality rate at 1 year was lower in the residual MR ≤ 1 group (10.3%) than in the MR 2 + group (18.9%; *p* < 0.001) and MR 3 + /4 + group (16.9%; *p* = 0.06). However, in the presence of advanced HF, defined in this study by the presence of LV dilatation or RV dysfunction, the survival benefit of residual MR ≤ 1 over MR 2 + was no longer detectable.

In our analysis, residual MR ≤ 1 + after M-TEER is associated with lower all-cause mortality in patients with both advanced and no-advanced HF. Thus, our study suggests that even patients with advanced HF may experience a benefit on mortality with an optimal MR reduction by TEER. Even though in advanced HF patients HF hospitalization and the composite outcome are only numerically lower in patients receiving an optimal MR reduction as compared to those who did not, advanced HF status seems not to affect the beneficial effect of optimal M-TEER on outcomes. Notably, the possible underestimation of the number of HF hospitalizations in the GIOTTO registry may also have affected the results.

Different definitions of advanced HF may have contributed to the different result of our analysis as compared to the OCEAN-Mitral study.

Notably, the rate of optimal M-TEER results is increasing because of continuous improvement in M-TEER technology and in operators’ experience. Indeed, it is currently possible to treat complex anatomies with a high rate of optimal result by using new-generation devices at high-volume centers [37]. Proper pre-procedural planning is crucial, including evaluating the MR mechanism, annular diameters and area, and leaflet length. Considering a strategy involving two or more devices and selecting the appropriate device size are also important [38]. 

Further research is needed to better establish the role of M-TEER in patients with advanced HF. The ongoing MITRAl regurgitation treatment in ADVANCEd Heart Failure (MITRADVANCE-HF) prospective, randomized, controlled, open-label, multicenter trial (NCT05292716) will enrol 190 patients with SMR and advanced HF who will be randomly assigned, in a 1:1 ratio, to a device arm consisting of MitraClip therapy added to optimal medical therapy (OMT) or a control arm of OMT alone. A composite hierarchical end-point including all-cause death, HF events, and quality of life changes assessed by KCCQ will be explored at 3-month follow-up.

## Limitations

Many limitations have to be acknowledged. First, this is an observational study with an intrinsic selection bias due to the lack of randomization. To partially overcome this issue, we compared patients with optimal M-TEER with those receiving a non-optimal M-TEER. Second, data were reported from different centers without independent adjudication of events. Thus, we used all-cause death as outcome of interest. Third, the reliability of the residual MR data could have been affected by the lack of a Core Laboratory and by the dynamic nature of SMR. Moreover, in the definition of advanced HF, data on exercise capacity were missing.

Finally, patients with SMR included in the GIOTTO registry should have been on optimal medical therapy. However, the rate of patients receiving ACE-i/ARBs/ARNIs was lower as compared with the current literature [5]. In addition, patients were recruited from 2016 to 2020, an era before establishment of SGLT2 inhibitors (SGLT2i) as a standard therapy for HFrEF patients.

## Conclusions

In a large real-world SMR population undergoing M-TEER, advanced HF at baseline was associated with higher mortality at 2 years after the procedure. However, an optimal result of M-TEER was associated with a lower mortality irrespective of the presence of advanced HF at baseline.

## Supplementary Information

Below is the link to the electronic supplementary material.

Supplementary file1 (DOCX 384 KB)
