# Supplementary material for: Impact of heart failure severity on the mortality benefit of mitral transcatheter edge-to-edge valve repair
Source: Clin Res Cardiol. 2024 Jul 24;114(7):836–45. doi: 10.1007/s00392-024-02490-7 (PMC12202617; doi:10.1007/s00392-024-02490-7)
Supplement: Supplementary file 1 — Supplementary file1 (DOCX 384 KB) [file 392_2024_2490_MOESM1_ESM.docx]

**SUPPLEMENTARY FIGURES LEGEND**

**Supplementary Figure 1.** **Estimated Kaplan Meier plots for HF hospitalization according to advanced HF.** Cumulative incidence of 2-year HF hospitalization in patients with and without advanced HF.

**Supplementary Figure 2.** **Estimated Kaplan Meier plots for all-cause mortality and HF hospitalization according to advanced HF.** Cumulative incidence of 2-year all-cause mortality and HF hospitalization in patients with and without advanced HF.

**Supplementary Figure 3. Estimated Kaplan Meier plots for HF hospitalization according to optimal M-TEER in no-advanced HF.** Cumulative incidence of 2-year HF hospitalization in patients who received or not an optimal M-TEER in no-advanced HF.

**Supplementary Figure 4. Estimated Kaplan Meier plots for all-cause mortality and HF hospitalization according to optimal M-TEER in no-advanced HF.** Cumulative incidence of 2-year all-cause mortality and HF hospitalization in patients who received or not an optimal M-TEER in no-advanced HF.

**Supplementary Figure 5. Estimated Kaplan Meier plots for HF hospitalization according to optimal M-TEER in advanced HF.** Cumulative incidence of 2-year HF hospitalization in patients who received or not an optimal M-TEER in advanced HF.

**Supplementary Figure 6. Estimated Kaplan Meier plots for all-cause mortality and HF hospitalization according to optimal M-TEER in advanced HF.** Cumulative incidence of 2-year all-cause mortality and HF hospitalization in patients who received or not an optimal M-TEER in advanced HF.

**Supplementary Figure 1.**

**Supplementary Figure 2.**

**Supplementary Figure 3.**

**Supplementary Figure 4.**

**Supplementary Figure 5.**

**Supplementary Figure 6.**
